# Influence of lipids on the hydrophobic barrier within the pore of the TWIK-1 K2P channel

**DOI:** 10.4161/19336950.2014.981987

**Published:** 2014-12-08

**Authors:** Prafulla Aryal, Firdaus Abd-Wahab, Giovanna Bucci, Mark SP Sansom, Stephen J Tucker

**Affiliations:** 1 Clarendon Laboratory, Department of Physics; University of Oxford; Oxford, UK; 2 Department of Biochemistry; University of Oxford; Oxford, UK; 3 OXION Initiative in Ion Channels and Disease; University of Oxford; Oxford, UK

**Keywords:** hydrophobic gating, K2P channel, lipid, Potassium channel, TWIK-1

## Abstract

Several recent ion channel structures have revealed large side portals, or ‘fenestrations’ at the interface between their transmembrane helices that potentially expose the ion conduction pathway to the lipid core of the bilayer. In a recent study we demonstrated that functional activity of the TWIK-1 K2P channel is influenced by the presence of hydrophobic residues deep within the inner pore. These residues are located near the fenestrations in the TWIK-1 structure and promote dewetting of the pore by forming a hydrophobic barrier to ion conduction. During our previous MD simulations, lipid tails were observed to enter these fenestrations. In this addendum to that study, we investigate lipid contribution to the dewetting process. Our results demonstrate that lipid tails from both the upper and lower leaflets can occupy the fenestrations and partially penetrate into the pore. The lipid tails do not sterically occlude the pore, but there is an inverse correlation between the presence of water within the hydrophobic barrier and the number of lipids tails within the lining of the pore. However, dewetting still occurs in the absence of lipids tails, and pore hydration appears to be determined primarily by those side-chains lining the narrowest part of the pore cavity.

## Introduction

Ion channels are dynamic transmembrane proteins that reside within phospholipid bilayers. Thus a full understanding of their functional properties not only relies upon our knowledge of the channel structure itself, but also on those interactions which occur between the channel and its lipid environment.^1-3^ There is now an increasing body of evidence that the interaction of membrane lipids with many classes of transmembrane proteins, including many ion channels, can profoundly influence their structure and function. Indeed, recent advances in X-ray crystallography,^4^ mass spectrometry^5^ and molecular dynamics simulations^6^ have now been able to define these interactions with a high degree of accuracy and prediction.

The overall structural organization of most ion channels means that the sites which interact with membrane lipids typically reside on the outer surfaces of the protein. However, recent structures of several sodium and potassium channels have revealed relatively large side portals or ‘fenestrations’ between the transmembrane helices of these proteins.^7-9^ These fenestrations connect the transmembrane pore to the lipid bilayer. In the case of the voltage-gated sodium channel the fenestrations are thought to be important for the access of lipophilic molecules such as local anesthetics into the ion conduction pathway.^9-11^

Fenestrations are also present in crystal structures of the K2P (*KCNK*) family of potassium channels.^7,8,12^ These channels contain 2 pore domains per subunit and assemble as dimers to form a pseudo-tetrameric K^+^ channel pore, thereby resembling other (tetrameric) potassium channels.^13^ However, unlike most tetrameric K^+^ channels they are not thought to gate via constriction of a cytoplasmic bundle-crossing gate.^14-17^ The 2 pore-lining helices (M2 and M4) are different in sequence and asymmetrical in their architecture. This asymmetry results in a gap at their interface that potentially exposes the central ion conduction pathway to the hydrophobic core of the lipid bilayer. Furthermore, in the crystal structure of TWIK-1, 2 tubular regions of electron density within the inner pore were attributed to alkyl chains, and it was proposed that these might represent co-purified lipids.^8^ This led to a hypothesis that lipid tails within this section of the inner pore of the related TRAAK channel might be involved in channel gating via their insertion into the fenestrations and steric obstruction of the ion conduction pathway.^18^ It was suggested that movement of lipid tails within the fenestrations might represent a mechanism for the direct sensing of mechanical tension in stretch-activated K2P channels.^18^

In order to understand the dynamic structural behavior of the TWIK-1 channel we recently performed molecular dynamics simulations of the TWIK-1 crystal structure in a phospholipid bilayer.^19^ Interestingly, we observed that the inner pore of the channel was highly hydrophobic, and therefore prone to dewetting which creates a hydrophobic barrier within the ion conduction pathway.^20^ The hydrophobic ‘cuff’ which forms this energetic barrier is comprised of leucine sidechains. We found that substitution of these residues with isosteric polar side-chains not only resulted in full wetting of the pore *in silico,* but also correlated with increased functional activity of the channel.^19^

We also reported that the fenestrations in TWIK-1 were dynamic and capable of closing during our simulations, but that when the fenestrations were open, the alkyl tails of the surrounding phospholipid bilayer were able to enter the fenestrations, though not far enough to sterically occlude the inner pore.^19^ Interestingly, similar observations have been reported for MD simulations of prokaryotic voltage-gated sodium channel structures where it was found that lipid tails could also penetrate the fenestrations.^10,21^

However, even without steric occlusion of the inner pore, the presence of lipid tails within the fenestrations might be expected to contribute to the hydrophobicity of the TWIK-1 inner pore cavity and so influence the dewetting process. Therefore, to investigate the contribution of the surrounding lipids to the hydrophobic barrier in the TWIK-1 pore, we have now carried out longer simulations of TWIK-1 in a POPC lipid bilayer. Here we describe the dynamics of the interactions between the channel protein, membrane lipids, and the hydrophobic barrier.

## Results and Discussion

### Two distinct fenestrations within TWIK-1

**Figure 1.**
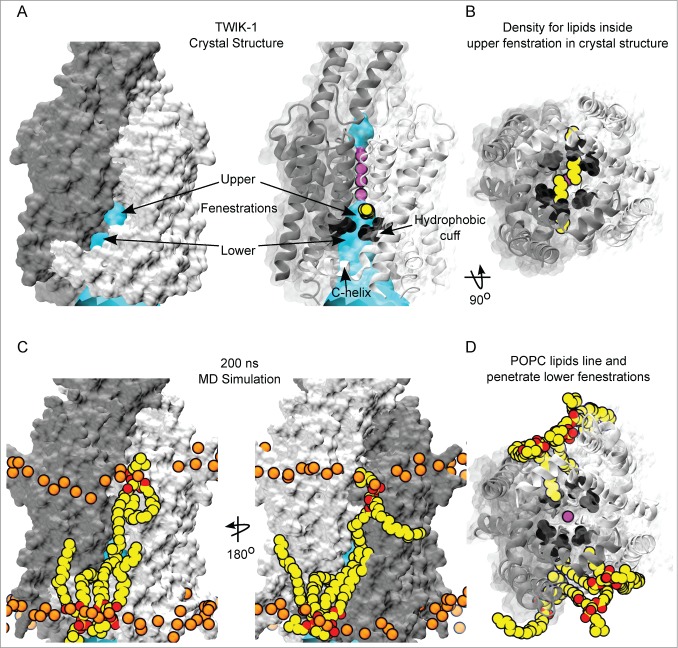
Interaction of lipids with the fenestrations of TWIK-1. (**A**) (left) Molecular surface representation of TWIK-1 crystal structure subunit interface shows 2 distinct gaps, termed upper and lower fenestrations. The channel is colored by subunit, with cyan HOLE surface depicting the ion conduction pathway. (right) Cartoon representation of the TWIK-1 crystal structure with side chain atoms of L146, L264 and L261 of the hydrophobic-cuff shown as black spheres. The upper fenestration is at the interface of pore-lining helices at the subunit interface, electron density attributed to alkyl chains (yellow) was found below selectivity filter (purple K^+^ ions) and above the hydrophobic cuff, whereas the lower fenestration is below the hydrophobic cuff. (**B**) A bottom up view of the inner-pore of the TWIK-1 crystal structure shows that the alkyl chains found inside the upper fenestrations block the ion conduction pathway. (**C**) Relative position of the POPC lipids on the subunit interface of TWIK-1 at the end of a 200ns MD simulation of the channel embedded in a POPC bilayer. TWIK-1 is shown as in A; Carbons atoms of POPC are colored yellow, oxygen red and nitrogen blue. Phosphorus atoms of the bilayer lipids are shown as spheres and colored orange for orientation. The upper fenestration is approximately at the center of the bilayer, and lipid tails from both the upper and lower leaflet can approach the upper fenestration, but do not penetrate the cavity. By contrast, lipid tails fully occupy the lower fenestrations. (**D**) A bottom up view of the pore at the end of the MD simulation showing lipid tails from the lower leaflet occupying both of the lower fenestrations, with one lipid tail penetrating the pore.

Molecular surface representations of the TWIK-1 crystal structure reveal that the fenestration at the interface between subunits can be divided into 2 distinct regions we term the ‘upper’ and ‘lower’ fenestrations (**Fig. 1A**). This division is formed by the side chains of L146 on M2 and L264 on M4 (**Fig. 1B**). Interestingly these residues, along with L261, contribute to the hydrophobic cuff that promotes dewetting of the inner pore.^19^ The upper fenestration is at the interface of the M2/M4 helices and is above the hydrophobic cuff. Tubular electron density was resolved in the crystal structure. This is thought to represent two 11-carbon alkyl chains penetrating the upper fenestrations and inner pore, (**Fig. 1B, C**).^8^ The lower fenestration is between the hydrophobic cuff and the C-helix at the cytoplasmic entrance to the pore (**Fig. 1A, B**).

The TWIK-1 structure was embedded in a POPC (1-palmitoyl-2-oleoyl-*sn*-glycero-3-phosphocholine) bilayer and 2 simulations run, each for 200 ns. In our previous study we observed dewetting of the inner pore irrespective of whether the backbone was restrained or not.^19^ Therefore, to ensure the fenestrations remained open and accessible throughout the simulations, a positional restraint was imposed on the Cα-atoms of the protein backbone. POPC was chosen because it represents a typical phospholipid containing both a saturated (palmitoyl, C16:0) and an unsaturated (oleoyl, C18:1) alkyl tail, and is routinely used for MD simulations of membrane proteins.

Interestingly, we found that throughout these simulations the upper fenestration was located at approximately the center of the bilayer. Consequently, tails of POPC lipids from both the inner and the outer-leaflet were able to line the subunit interface and upper fenestration, but were not long enough to fully penetrate and occlude the inner cavity (**Fig. 1C**). This suggests that direct lipid block of the inner pore is unlikely. However, some mammalian saturated phospholipids have tails up to 24 carbons in length,^22^ and it remains possible that such longer phospholipid tails might penetrate further through the upper fenestration. It is interesting to note that the TWIK-1 crystal structure was solved using protein isolated from *Pichia pastoris* and that yeast contain very long (26:0) phosphosphingo-lipids.^22,23^

In marked contrast however, the lower fenestration is located near the middle of the inner leaflet, making it a more accessible portal for lipid tail entry (**Fig. 1A, C**). We observed that lipids tails from the inner leaflet occupied the lower fenestrations throughout the entire duration of the 200 ns simulation. Both the unsaturated oleoyl and the saturated palmitoyl lipid tails of POPC were able to occupy this fenestration. Lipid tails were capable of entering the inner cavity just below the hydrophobic cuff. However, they did not penetrate far enough to occlude the ion conduction pathway (**Fig. 1D**).

### Influence of lipids on the hydrophobic barrier

Our previous study demonstrated that dewetting of the hydrophobic barrier deep within the inner pore of TWIK was dependent upon the relative hydrophobicity of the surface lining the pore.^19^ Consequently, if the alkyl tails of the lipids are capable of entering the fenestrations and penetrating into the cavity then it might be anticipated that they could also influence the hydrophobic barrier in TWIK-1. We therefore next examined the correlation between the presence of such lipid tails and the behavior of water within the hydrophobic cuff.

**Figure 2.**
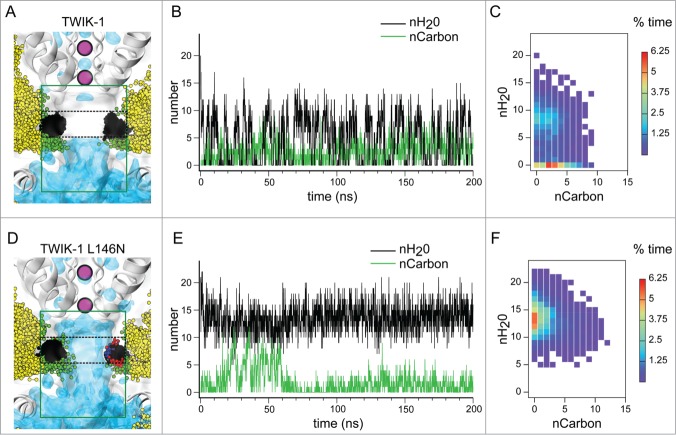
Influence of lipid tails in the ion conduction pathway of TWIK-1. (**A**) Average water density from a 200ns simulation inside the TWIK-1 ion conduction pathway. Snapshots (taken every 1ns) of lipid residues lining the pore, as well as the snapshots of the L146 and L264 sidechains from both subunits, are depicted as ball and sticks. Lipid carbon atoms lining and entering the pore (as defined by the green box) are colored green, whereas carbons outside of the lining are colored yellow. Carbons of the L146 and L264 side-chains are shown in black. Black dashes indicate the region within the green box used to count the number of waters within the hydrophobic cuff. The selectivity filter containing purple K^+^ ions and C-helix at the start of the simulation are shown for orientation. (**B**) Number of water molecules within the hydrophobic cuff (black) taken every 0.1 ns during a 200 ns MD simulation. Also shown are the number of lipidic carbon atoms (green) which enter the pore cavity as defined by the green box in panel A. (**C**) Correlation heat map for the 2 traces shown in panel B. Data shown as percent of time. (**D–F**) As above but for the L146N mutant channel. Oxygen and nitrogen atoms colored red and blue respectively.

Consistent with our previous studies, we observed dewetting of the hydrophobic inner pore of TWIK-1 within the first few nanoseconds of the simulation and this was concurrent with lipid tails entering the fenestration and thereby forming part of the lining of the pore (**Fig. 2A and B**). We also observed liquid-vapor transitions and fluctuating lipid penetration into the pore lining throughout the simulations (**Fig. 2A and B**).

We next compared the number of water molecules within the hydrophobic cuff, with the number of lipid carbon atoms present in the pore and its lining (**Fig. 2B**). Although both processes were subject to fluctuations, their time series suggests some correlation between dewetting of the hydrophobic barrier and the presence of lipid tails exposed to the pore. We therefore generated a correlation heatmap; this shows that although dewetting can occur when lipids are completely absent from the cavity, the highest instance of dewetting occurs when 2 or 3 lipid tail carbons are present in the mouths of the fenestrations which line the central cavity (**Fig. 2C**).

Our previous study demonstrated that dewetting of the cavity was profoundly influenced by the hydrophobicity of residues within the hydrophobic cuff. For example, the L146N mutation was found to induce full hydration of the inner cavity.^19^ We therefore examined 2 identical simulations of the L146N mutant channel structure. Interestingly, we observed that during the first 60 ns of the simulation up to 12 lipid carbon atoms penetrated into the cavity of the L146N mutant, but the cavity remained hydrated throughout the entire simulation (**Fig. 2D, E**). Furthermore, we found a decrease in the percent occupancy of carbons in the pore (35% for the L146N mutant vs 55% for WT in two 200ns simulations) (**Fig. 2F**). Therefore, polar substitutions in the hydrophobic cuff not only prevent dewetting of the cavity, but also decrease the overall occupancy of lipids.

In summary, these simulations demonstrate that the terminal carbons of the surrounding lipids can penetrate the fenestrations of TWIK-1 and contribute to the lining of the inner cavity, but not far enough to sterically occlude the pore. The presence of lipids within the fenestrations also influences the relative hydrophobicity of the surface lining the inner pore, and dewetting occurs most frequently when lipid tails line the cavity. However, dewetting still occurs in the absence of such lipid tails, and it is the relative hydrophobicity of residues at the narrowest constriction point (i.e. the hydrophobic barrier) that primarily determines the hydration status of the inner pore.

Future simulations with more physiological lipid head-group compositions, and mixed lipid tail length are clearly needed to fully understand the behavior of TWIK-1 and other K2P channels in complex bilayers. Hydrophobic lipid tails might still regulate TWIK-1 channel activity by influencing this hydrophobic barrier. However, only long lipid tails (e.g. ≥24 carbon^22^) might be able to directly occlude the inner pore. TREK-1 and TRAAK channels retain their mechanosensitivity when reconstituted into a bilayer consisting primarily of 16 and 18 carbon lipid chains.^24^ Therefore, mechanogating by dynamic modulation of a lipid block within the conduction pathway appears unlikely. Nevertheless, it remains possible that the presence of lipid tails within the fenestrations might influence the biophysical properties of ion conduction, as well as more global conformational changes involving movement of the transmembrane helices and closure of the fenestrations.^12,25^

In complex cellular membranes, K2P channel function is clearly affected by the composition of the surrounding lipids,^26-28^ and lipid composition can vary dramatically between different intracellular organelles.^23^ It is therefore possible that the interaction of different physiological lipids, and even lipophilic drugs with these fenestrations remains a potential mechanism for the control of K2P channel activity.

## Methods

Molecular dynamics simulations were similar to those described previously.^19^ Briefly, missing atoms and loops were modeled into the TWIK-1 crystal structure (PDBID: 3UKM).^8^ To embed the protein into a bilayer, the structure was converted into coarse grain representation (Martini v2.1) and embedded into a POPC bilayer by running coarse grain self-assembly and equilibration simulations for 500 ns. The resultant protein embedded in a bilayer containing 212 POPC lipids was then converted into atomistic structure using the CG2AT method.^29,30^ Atomistic simulations reported here employed GROMOS 53A6 with SPC water and 150 mM KCl. K^+^ ions were placed at positions S2 and S4 in the selectivity filter with an additional ion in the inner pore. Two water molecules were also added to the filter at the S1 and S3 positions. Then 200 ns equilibration simulations were run at constant pressure (1 atm) and temperature (310K) with Cα atoms of the protein restrained with spring constant of 10 KJ mol^−1^ Å^−2^. The simulations were repeated by randomizing the initial velocity to obtain two 200 ns simulations for TWIK-1 WT protein. Two additional 200 ns simulations were run for the L146N *in-silico* mutant, where both of the L146 residues of the dimer were mutated to asparagine using pyMOL mutagenesis script on the initial TWIK-1 WT system. HOLE radius profiles were generated using MD analysis and HOLE.^31^ Average water density maps were generated by using the Volmap plugin tool with 3-dimensional grids every 0.5 Å for each simulation. The maps were then normalized to bulk water density and visualized at an isovalue of 0.5. Water occupancy at the hydrophobic constriction was analyzed by counting the number of water oxygen atoms in a box of 20 × 20 × 5 Å located −5 Å<z<−10 Å below the S4 ion binding site (Thr117 and Thr225, which define the 0 Å position on the *z*-axis, whereas the number of carbon atoms were counted in a box 20 × 20 × 20 Å below the S4 ion binding site using vmd tcl script.
